# A Cancer Center’s Approach to Engaging African American Men About Cancer: The Men’s Fellowship Breakfast, Southeastern Michigan, 2008–2014

**DOI:** 10.5888/pcd11.140187

**Published:** 2014-09-25

**Authors:** Aisha T. Langford, Derek M. Griffith, Derrick D. Beasley, Effat Id-Deen Braxton

**Affiliations:** Author Affiliations: Derek M. Griffith, Vanderbilt University Institute for Research on Men’s Health, Nashville, Tennessee; Derrick D. Beasley, Centers for Disease Control and Prevention, Atlanta, Georgia; Effat Id-Deen Braxton, University of Michigan, Ann Arbor, Michigan. Dr Langford is also affiliated with VA Health Services Research and Development Service, Ann Arbor, Michigan.

## Abstract

**Background:**

Despite disproportionate rates of cancer morbidity and mortality among African American men, few community-based efforts have been developed and sustained to educate African American men about cancer. The University of Michigan Comprehensive Cancer Center implemented a series of breakfasts to improve cancer awareness, screening, and education among African American men. This article describes the rationale for and history of the community intervention.

**Community Context:**

The 21 breakfasts were held from 2008 through mid-2014 in Ypsilanti and Ann Arbor, Michigan. Ypsilanti ranks below Michigan and the nation on most socioeconomic indicators, although most residents are high school graduates (88% in Ypsilanti and 96.5% in Ann Arbor). African American men in Ypsilanti have higher death rates for diseases associated with poor diet and inadequate physical activity compared with Ypsilanti whites and general populations in Michigan and the nation.

**Methods:**

We conducted a multicomponent qualitative process evaluation including staff meetings, conversations with participants, and focus groups. We collected 425 post-event surveys to evaluate the breakfasts quantitatively.

**Outcomes:**

Participants were African American (85%), were aged 51 to 70 years (54%), had health insurance (89%), and had some college education (38%). Fifty-three percent of participants reported interest in the breakfast topics including nutrition; 46%, prostate cancer; 34%, colorectal cancer, and 32%, pain management; 62% reported willingness to participate in a clinical trial.

**Interpretation:**

African American men are interested in learning about health and are willing to attend a health-focused breakfast series. The Men’s Fellowship Breakfast is a promising strategy for bringing men together to discuss cancer screening and risk reduction.

## Background

Disparities in cancer mortality rates between African Americans and whites have widened over the years for all cancers combined and for most major cancers (1). Men have slightly less than a 1 in 2 lifetime risk of developing cancer, and that risk increases dramatically for African American men (2). According to the American Cancer Society, the death rate for all cancers combined is 33% higher among African American men than among white men (3). For African American men aged 45 years or older, cancer is the first or second leading cause of death after heart disease (4,5). Although several factors contribute to cancer disparities among African American men, including lack of health insurance, later-stage diagnosis, differential access to quality cancer care, and socioeconomic position (2,6), the limited number of targeted health education programs for this group may further complicate the problem. In particular, the sociocultural factors that may affect the health of African American men are often overlooked (7–9).

Cancer education efforts to improve the health knowledge of African American men have largely focused on prostate cancer screening and, to a lesser extent, colorectal cancer screening, physical activity, hypertension, obesity, diabetes, and fruit and vegetable consumption (10–13). However, little has been done to investigate the best strategies (including content and mode of delivery) for improving awareness, knowledge of cancer risk reduction, and health-promoting behaviors among this group. Although some educational initiatives exist to improve African American men’s health knowledge and access to health services (eg, Black Men Speak, Inc. [Oakland, California], Men of Color Health Awareness [Springfield, Massachusetts], Project Brotherhood [Chicago, Illinois], Black Men’s Health and Wellness Expo [Orlando, Florida]), few initiatives meet regularly and address the spectrum of determinants of cancer among African American men. Educational programs delivered through faith-based organizations and barbershops have also been used to reach African American men (14,15), but these programs tend to be time-limited and focused on a narrow range of topics.

The Men’s Fellowship Breakfast (MFB) was created by the University of Michigan Comprehensive Cancer Center to provide evidence-based, timely health information for men in southeastern Michigan and in a safe, interactive, nonjudgmental environment. The MFB began as an exploratory effort to identify a strategy for engaging men about cancer risk reduction and health promotion behaviors. The purpose of this article is to describe the first 6 years of MFB activities, provide details on MFB as a model for health education, and share our process for program planning, which can be used to develop other community-based interventions. Additionally, we report on the demographic characteristics of participants and the responses from postevent surveys.

## Community Context

Our population of interest is lay community men from cities in southeastern Michigan, the 21 breakfasts were held in Ypsilanti (2008–2011) and Ann Arbor (2012–2014). The MFB took place in the Ypsilanti metropolitan statistical area (MSA), the fifth largest MSA in Michigan. This MSA includes the cities of Ypsilanti and Ann Arbor. Ypsilanti ranks below Michigan and the nation on most socioeconomic indicators (16,17), although most MSA residents are high school graduates (88% in Ypsilanti and 96.5% in Ann Arbor). The Ypsilanti MSA is approximately 33% African American, whereas the surrounding area is predominantly white (17). African American men in Ypsilanti have higher death rates than Ypsilanti whites and the general populations in Michigan and the nation for diseases associated with poor diet and inadequate physical activity (18,19).

The concept of the breakfast was inspired by earlier attempts at men’s health events and our ongoing work with African American churches through Body and Soul, a national program to promote healthy eating and physical activity among African Americans. For example, many African American churches have a men’s ministry that, as part of their regular activities, hosts prayer or fellowship breakfasts. We identified these breakfasts as historically grounded and culturally familiar. We assumed that men of faith (and perhaps other men in the community) would be comfortable participating in a public version of a men’s fellowship breakfast. Consequently, we piloted a health-focused, community-wide breakfast in May 2008 that was free and open to all men. Other models were used in our area to reach African American men with some success (eg, barbershop outreach, prostate cancer screenings, exercise programs); however, many of these efforts were part of time-limited research projects, were annual events, or attracted small numbers of African American men. We wanted a model that was sustainable and responsive to the needs of African American men in our community.

The MFB is the primary vehicle of the University of Michigan Comprehensive Cancer Center for reaching African American men in southeastern Michigan. The objectives of the MFB are 1) to educate men about cancer prevention, screening, and treatment options; and 2) to create a safe, comfortable space in the community for men to talk with peers and professionals about general health and wellness topics and other social concerns. Outcomes of interests include improved knowledge about cancer, age-appropriate health screenings, and lifestyle factors that affect risk for developing chronic disease.

## Methods

### Format of the breakfasts

Before the MFB series, our first attempt at a men’s health program open to the community was in November 2007: Spotlight on Men’s Health. It was held at a local community center on a weekday evening. The event was free, dinner was served, and topics included prostate cancer, obesity, physical activity, and gender-role strain (20). Although we observed good discussion and positive interaction among participants, the attendance was much lower than expected (12 people attended; 8 men, 4 women). It was later brought to our attention that the day of the week (Thursday), time (6:30–8:00 PM), and location (a community center) may have been a problem; thus, the idea of hosting a Men’s Fellowship Breakfast on a Saturday morning at a centrally located space was born.

The MFB started as a one-time experiment to determine whether men would attend a community health event. Before hosting the first breakfast, we informally asked African American men in the community and African American male colleagues about what they thought were the most pressing health issues affecting African American men in our area. Our initial plan was to offer the breakfast once annually; however, after the first breakfast, several men requested that we host the breakfast regularly because few, if any, community efforts existed to address the health needs of African American men. We decided to hold the MFB 3 times per year on Saturday mornings from 8:30 to 11:00. We hypothesized that Saturday mornings would be a good time to convene men because it did not conflict with other community meetings and still allowed men to have most of their day to take care of other business. We purposely included the word “fellowship” in the title of our event because we wanted to connect these efforts to our work with African American churches and to convey a sense of community and friendship. Building on previous work with the faith community, we contacted men’s ministries at local churches. Some of the ministries held their men’s meetings, Bible study, and prayer right before our event in the same room we used.

The time, day of the week, and location of each breakfast was the same, but the topics and speakers changed. For the first 3 years (10 events in 2008 through 2010), the breakfasts took place in the ballroom of a local hotel; they then took place (11 events in 2011 through June 2014) in a less formal and less expensive conference center at a local community college. Both venues are popular, centrally located spaces for group events in Ann Arbor and Ypsilanti. As we had hoped, the men preferred the less formal community college setting. Depending on the topics and speakers, breakfasts included PowerPoint presentations, panel discussions, keynote speakers, small group exercises, health fairs, or cooking demonstrations.

Since the first MFB, men have reported they found the question-and-answer portion to be the most helpful aspect of the event; thus, we decided to let audience questions drive the discussion. That is, instead of asking speakers to give a comprehensive lecture, we asked them to give a “big picture” overview to get the conversation started. For example, rather than asking a speaker to provide details on prostate cancer gene mutations, we asked the speaker to explain *why* genes are relevant to prostate cancer and *how* genes can provide useful information about a particular type of prostate cancer, including which treatment option may work best. For topics covered more than once (eg, prostate cancer), we suggested that speakers describe emerging topics in the field. For example, when the US Preventive Services Task Force advised against mass prostate-specific antigen screening in 2011, we discussed the pros and cons of the screening as well as what the revised guidelines meant for African American men, who have the highest rates of prostate cancer.

### Planning team

We met regularly with a small planning team of 4 African American men ranging in age from 28 to 70 to get feedback on topics, potential speakers, and marketing. Members of the planning team were residents of our target communities and included an assistant professor specializing in African American men’s health, a retired manager in the automobile industry, an elder from a local church, and a personal trainer; the intent was to create a planning group that could draw on different life experiences and areas of expertise. Most importantly, they all interacted with other men in the community in different capacities and were committed to creating a social norm of wellness for African American men.

### Topics and speakers

We proactively solicited ideas from men who regularly participated in the breakfasts and from men in the community who had never attended a breakfast. This dialogue generated ideas for topics and shed light on where we should advertise to recruit a wider range of men. We encouraged men to share their ideas through in-person conversations, telephone calls, e-mail correspondence, and surveys. Additionally, we asked for anonymous feedback on note cards. The note card feedback alerted us that many men were struggling with depression, marital issues, and sexual dysfunction. Thus, although each breakfast featured some aspect of cancer prevention, screening, or treatment, we also included topics beyond physical health (Table 1).

**Table 1:** Men’s Fellowship Breakfast Topics by Date, Attendance, and Type of Speaker, Southeastern Michigan, 2008–2014

Date	Topic	Attendance, No.	Type of Speaker
5/17/08	Prostate cancer	125	Oncology nurse, prostate cancer survivor, assistant professor of public health
9/13/08	Prostate and colon cancers	100	Urologist and colorectal surgeon
1/10/09	Diet, weight, and exercise	90	Assistant professor of public health, personal trainer, registered dietitian
5/2/09	Lung and oral cancers	75	Dentist, thoracic oncologist, professor of dentistry
8/29/09	Sexual function and physical activity	115	Sexual function counselor, personal trainer
12/15/09	Stress, nutrition, and sexual function	100	Sexual function counselor, registered dietitian, Tai Chi master
5/15/10	Weight loss and colon cancer	105	Gastroenterologist, former contestant from NBC’s The Biggest Loser
6/26/10	Men’s Health and Fitness Day (screenings, panel, games, Ask the Experts)	90	College football coach, college baseball coach, lawyer, primary care physician
8/28/10	Prostate cancer and clinical trials	140	Urologists, former NFL player
12/18/10	Depression and nutrition	85	Former NFL player, registered dietitian
4/9/11	Men’s health, estate planning, and physical activity	100	Family medicine physician, attorney, personal trainer
8/28/11	Prostate cancer, research, and physical activity	95	Urologist, assistant professor in public health
12/17/11	Getting your mind right for the new year	75	Director of athletic counseling
5/19/12	Healthy eating, colon health, and cooking demonstration	95	Gastroenterologist, registered dietitian, executive chef
8/25/12	Weight loss, finances, and estate planning	105	Internal medicine physician, certified financial planner, lawyer
12/15/12	Pain management, mental health, and relationships	110	Internal medicine physician, psychiatrist, cognitive behavioral therapist
4/6/13	Heart disease, cancer, and self-motivation	105	Internal medicine physician, nurse, kidney recipient
9/21/13	Prostate cancer and sexual dysfunction	130	Sexual function counselor, urologist
12/15/13	Communication with your health care provider and spouse; motivation to take charge of your health	90	Sexual function expert, director of athletic counseling
3/29/14	Colon cancer, cancer prevention/screening, and the ACA	80	Gastroenterologist, cancer researcher, and patient financial services counselor to help men enroll into the ACA
6/14/14	Bone, muscle and joint injuries; spirituality and health; cancer prevention and screening	105	Primary care physician, clergy member of health system’s pastoral care team, and oncology nurse

Abbreviations: NBC, National Broadcasting Company; NFL, National Football League; ACA, Affordable Care Act.

Speakers included primary care physicians, oncologists, public health professionals, nurses, researchers, social workers, dietitians, and cancer survivors. Per the suggestion of participants to focus on the “total man” and not just physical health, we featured lawyers, behavioral therapists, financial counselors, college coaches, and motivational speakers. The range of topics highlighted the importance of recognizing the broad way in which men define health and the determinants of health. An honorarium of up to $250 was given to speakers to help cover the cost of transportation and time spent outside of normal work hours preparing for and participating in the event.

### Funding

There was no charge to men who attended the breakfasts; all of our community outreach events are free to the public and funded by the University of Michigan Comprehensive Cancer Center. For approximately $2,000 per breakfast, we reached 80 to 150 men per event. This cost estimate included food, printing of flyers and educational brochures, and honoraria.

### Recruitment

The first MFB was promoted among local African American churches, faculty and staff members at local universities, hospitals, and social service agencies. As the breakfasts continued, we expanded our marketing strategies to include press releases, paid newspaper and radio advertisements, and distribution of postcards at community events. We also distributed flyers to barbershops, beauty salons, civic organizations, fraternities, sororities, and minority-owned businesses. Flyers and information about the breakfasts were also posted to our website (www.mcancer.org/outreach) and Facebook page (www.facebook.com/UniversityofMichiganComprehensiveCancerCenter); both options are free and maintained by our marketing department. We also used paid advertising in the local mall (eg, signage in food court and near men’s stores). Our most effective marketing strategies were e-mail blasts to past participants and word-of-mouth invitations.

### Participation of women

Although the breakfasts are not closed to women (usually <5 women attend), women are not encouraged to attend. E-mails blasts usually include the following message: “The breakfast is free and open to all men.” The decision to keep the event focused on men was born out of a desire to create a safe, nonjudgmental space for men to discuss often-sensitive issues related to physical, emotional, and mental health. For example, some topics covered relationship challenges, communication with one’s partner, difficulty in fulfilling socially important roles (eg, provider, father), and sexual function. Each topic prompted rich conversations that may not have been so candid if women, particularly wives, had been present. However, we hosted other events that were open to men and women and focused on breast, colon, and prostate cancers.

## Outcomes

The process evaluation for the breakfasts was done primarily in 2 ways: pen-and-paper surveys and focus groups.

### Survey feedback

At 5 breakfasts during 2008–2010, we collected 425 self-report surveys. The survey, completed at the end of each breakfast, asked questions about demographics, future topics of interest, and how men had heard about the breakfasts. The survey also provided the opportunity to write in comments or suggestions. We collected survey data at subsequent breakfasts, but in this article, we report on data collected only during 2008 through 2010 for 2 reasons: 1) this period had the most dynamic change in topics and format, and 2) we had the most complete data from this period. Most survey respondents (Table 2) were African American (85%), were aged 51 to 70 years (54%), and had health insurance (89%). Of those responding to the question on the highest level of education attained, the greatest percentage reported some college (38%). The topics of most interest were nutrition (53% of survey respondent reported interest), prostate cancer (46%), colorectal cancer (34%), and pain management (32%). Approximately 71% of participants had heard of a clinical trial, 33% had participated in a clinical trial, and more than half were somewhat willing (38%) or strongly willing (24%) to participate in a clinical trial. The top 2 motivations for attending the event were “wanted to hear speaker” (30% of participants) and “friend or loved one affected by an illness” (26% of participants). Finally, 59% reported that their “top” source of health information is a health care provider; 41% reported their top source is a friend or relative.

**Table 2:** Results of Surveys (N = 425) Administered After 5 of 11 Men’s Fellowship Breakfasts, Southeastern Michigan, 2008–2010

Characteristic	Percentage
**Race/ethnicity**	
White	9
Black	85
Other	6
**Top reasons for attending the event**	
Friend or loved one affected by an illness	26
Wanted to hear speaker	30
Other	44
**Top places where you get health information**	
Friend or relative	41
Health care provider	59
**Do you have health insurance?**	
Yes	89
**Age group, y**	
<50	36
51–70	54
≥71	8
No response	2
**Highest level of education**	
Less than high school graduate	4
High school graduate or GED	18
Some college	38
College degree or more	36
No response	4
**Heard of a clinical trial**	
Yes	71
**Participated in a clinical trial**	
Yes	33
**Willing to participate in a clinical trial**	
Strongly unwilling	3
Somewhat unwilling	6
Neutral	18
Somewhat willing	38
Strongly willing	24
No response	11
**Topics most interested in for future programs^a^**	
Nutrition	53
Prostate cancer	46
Colorectal cancer	34
Pain management	32
Cancer and aging	27

Abbreviation: GED, general educational development.

a Participants could choose more than 1 response.

Other key outcomes were sustained attendance at the breakfasts, increased knowledge about cancer screening, and anecdotal reports of men going to the doctor for check-ups and colorectal cancer screenings.

### Focus group feedback

From 2011 through 2013, we held 3 focus groups to help shape the selection of future breakfast topics, speakers, and themes; each had 6 to 10 men (for a total of 26). Participants were diverse in age, work status, profession, life events, and educational attainment. Focus group discussions showed that participants had interest in the following topics: 1) physical activity as a part of one’s life, 2) affordable and accessible health care, 3) violence prevention, 4) relationships, 5) environmental stressors, 6) stress in the household, 7) overall stress management, and 8) financial planning.

## Interpretation

Our experience to date with the MFB showed that African American men care about cancer and other health issues. Our conceptual model (Figure) for the MFB was informed by models of stress and coping, the health belief model, and definitions of social support (21). The breakfasts provided social, informational, emotional, and appraisal support and cues to action. Regular attendance during 6 years and 21 breakfasts indicates that African American men want health information and will attend events when they are consistent and relevant. Therefore, it is incumbent upon health professionals to develop programs that are relevant, community-focused, and accessible to African American men. Such efforts will require that health care professionals engage, listen to, and serve as a resource for African American men on an ongoing basis, not only when they need something from the community (eg, clinical trial participants, donations).

**Figure:**
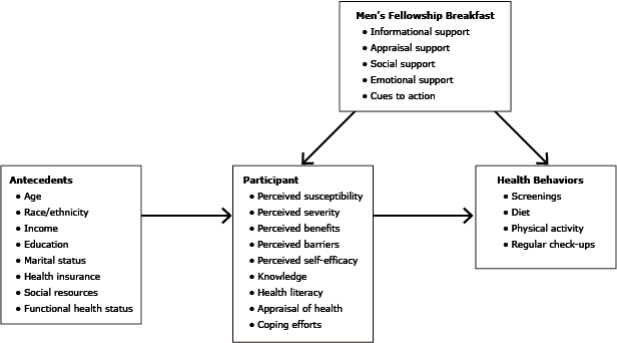
Conceptual model for Men’s Fellowship Breakfast, southeastern Michigan, 2008–2014.

We regularly communicate how we use the men’s ideas to shape the breakfasts. For example, at the beginning of each breakfast, we explain why we picked the topics for the day and how their feedback helped guide that decision. When we are not able to address a specific concern in the group setting, we follow up directly with the individual who raised the concern. For example, 1 participant had sleep apnea, so we connected him with our health sciences librarian, who in turn mailed him information about sleep apnea and sleep studies within a 40-mile radius of his home. Another man’s daughter was diagnosed with breast cancer and requested information on her behalf. We shared brochures, information about African American oncologists specializing in breast cancer (per his request), and the number to a toll-free cancer information service.

Directions for future breakfasts include expanding the topics to include other chronic conditions (eg, heart disease) and social factors that can affect health (eg, caregiving, unemployment). Future assessment will also include tracking preventive health and screening behaviors over time, which should be easier with the implementation of the Affordable Care Act.

We also plan to feature success stories on our website or at the breakfasts. For example, a participant lost 20 pounds in 1 year, and another participant got his colonoscopy after a 2-year delay. Although we realize that a men’s breakfast may not work for all communities, we have found these breakfasts to be a useful and efficient strategy for bringing men together to discuss cancer and other health issues.
